# *Kandelia obovata* afforestation enhances coastal soil quality over *Spartina alterniflora* invasion but increases phosphorus limitation

**DOI:** 10.1016/j.isci.2026.116315

**Published:** 2026-06-11

**Authors:** Yun Zhang, Shuangshuang Liu, Jinwang Wang, Xing Liu, Wenzhen Xin, Xiang Lu, Huizi Liu, Qiuxia Chen, Sheng Yang

**Affiliations:** 1 Zhejiang Institute of Subtropical Crops, Wen Zhou, Zhejiang 325005, China; 2 Wenzhou Key Laboratory of Resource Plant Innovation and Utilization, Zhejiang Institute of Subtropical Crops, Wenzhou, Zhejiang 325005, China; 3 Engineering Research Center for Southeast Coastal Characteristic Plants of National Forestry and Grassland Administration, Wenzhou, Zhejiang 325005, China; 4 College of Forestry, Beijing Forestry University, Beijing 100080, China

**Keywords:** Environmental management, Environmental science, Plant ecology, Soil chemistry, Soil ecology, Soil science

## Abstract

This study evaluated 0–100 cm soil properties among native *Carex scabrifolia*, invasive *Spartina alterniflora*, restored *Kandelia obovata*, and mixed stands of *K*. *obovata* and *S*. *alterniflora* in Oujiang Estuary, and among four *K*. *obovata* restoration sites in Zhejiang Province, China. Results showed vegetation type and soil depth significantly influenced soil properties. *K*. *obovata* soils displayed the lowest C:N and highest N:P ratios, total nitrogen, and available phosphorus among vegetation types, suggesting nitrogen sufficiency but phosphorus limitation. Soil attributes in *K*. *obovata* forests varied significantly by location and soil depth. Principal-component analysis ranked vegetation types as *K*. *obovata* > *S*. *alterniflora* > mixed stands> *C*. *scabrifolia*; and *K*. *obovata* sites as Yanpu Bay > Ximen Island > Oujiang Estuary > Dongshan Wharf. Therefore, *K*. *obovata* afforestation enhances soil quality, with Yanpu Bay and Ximen Island as optimal restoration areas. Restoration increases phosphorus demand, necessitating targeted supplementation for long-term ecosystem health.

## Highlights

• Vegetation type and soil depth significantly influence soil properties
• *Kandelia obovata* restoration improves soil quality over *Spartina alterniflora* invasion
• *Kandelia obovata* restoration suggests nitrogen sufficiency but phosphorus limitation

## Graphical abstract

**Figure:**
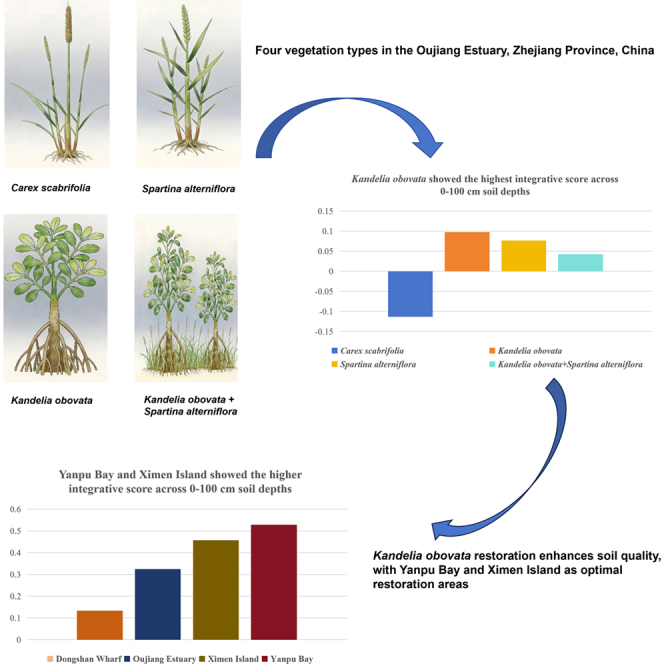

## Introduction

Wetland ecosystems exhibit a substantial carbon sequestration capacity and are of critical importance in maintaining ecological equilibrium, preserving biological diversity, regulating climatic factors, and facilitating landscape evolution.^1^^,^^2^ These ecosystems are often recognized as the “blue carbon sinks”.^1^^,^^2^ Among coastal wetlands, mangrove forests stand out as highly productive blue carbon ecosystems.^3^^,^^4^ Moreover, the restoration of mangrove is also acknowledged for having the potential to enhance coastal blue carbon storage.^5^^,^^6^^,^^7^

*Spartina alterniflora* was introduced to China from the United States. It is now regarded as a global invasive plant species and is frequently detected in coastal wetlands across China.^8^ The intrusion of *S*. *alterniflora* presents a substantial menace to the survival of the indigenous wetland ecosystem,^9^^,^^10^ leading to a decrease in habitat quality and biodiversity^11^ and causing the degradation of native ecological functions.^12^ The soil nutrient content, particularly carbon (C), nitrogen (N), and phosphorus (P), holds a significant position in biogeochemical cycles^13^^,^^14^ and serves as key indicators of soil nutrient status, material and energy fluxes, and ecosystem elemental balance.^9^^,^^15^ The invasion of *S*. *alterniflora* is a primary driver of altered soil nutrient dynamics in coastal marshes and mangroves,^6^^,^^16^^,^^17^ which in turn affects soil carbon storage, especially in mangrove areas.^18^ A number of research efforts have suggested that the invasion of *S*. *alterniflora* has led to a significant decline in the soil total nutrient reservoir of the mangrove ecosystems; moreover, the decrease of the carbon reservoir has substantially weakened the carbon sequestration capacity of coastal wetland ecosystems.^19^^,^^20^ A meta-analysis further indicated that the dominant species mainly affected the impacts of the *S*. *alterniflora* invasion on the content of SOC.^21^

*S*. *alterniflora* exhibits greater competitiveness and salt tolerance compared to native plants.^12^ Consequently, it is essential to manage the expansion of *S*. *alterniflora* to preserve the well-being and equilibrium of the coastal wetland ecosystems. *Kandelia obovata*, a true mangrove species that is most extensively distributed and the cold-resistant mangrove plant in China.^22^^,^^23^ It was introduced to Zhejiang Province in 1957^24^ and is currently utilized to manage the proliferation of *S*. *alterniflora*.^25^ The coastal wetlands of Zhejiang represent the northernmost distribution limit of mangroves in China, making them a critical ecological frontier.^26^ Studying mangrove restoration in the marginal habitat provides unique insights into mangrove resilience under extreme environmental pressures. Nevertheless, the studies on controlling of *S*. *alterniflora* invasion through the restoration of *K*. *obovata* has predominantly concentrated on the macrobenthos communities,^27^^,^^28^^,^^29^^,^^30^^,^^31^ carbon and nitrogen stocks and sources,^17^ microbial community,^25^^,^^30^ and carbon sink potential.^32^^,^^33^^,^^34^ A clear understanding of the soil properties among different vegetation types and across soil depths underpins effective strategies for maintaining soil quality, enhancing carbon sequestration,^35^ and preserving ecosystem well-being.^36^ However, it remains unclear how soil properties differ among invasive *S*. *alterniflora*, restored *K*. *obovata*, mixed stands of *S*. *alterniflora* and *K*. *obovata*, and the native salt marsh vegetation (*Carex scabrifolia*), particularly across the 0–100 cm soil profile. Furthermore, variations in soil characteristics across different restoration sites are still poorly understood.

**Figure 1:**
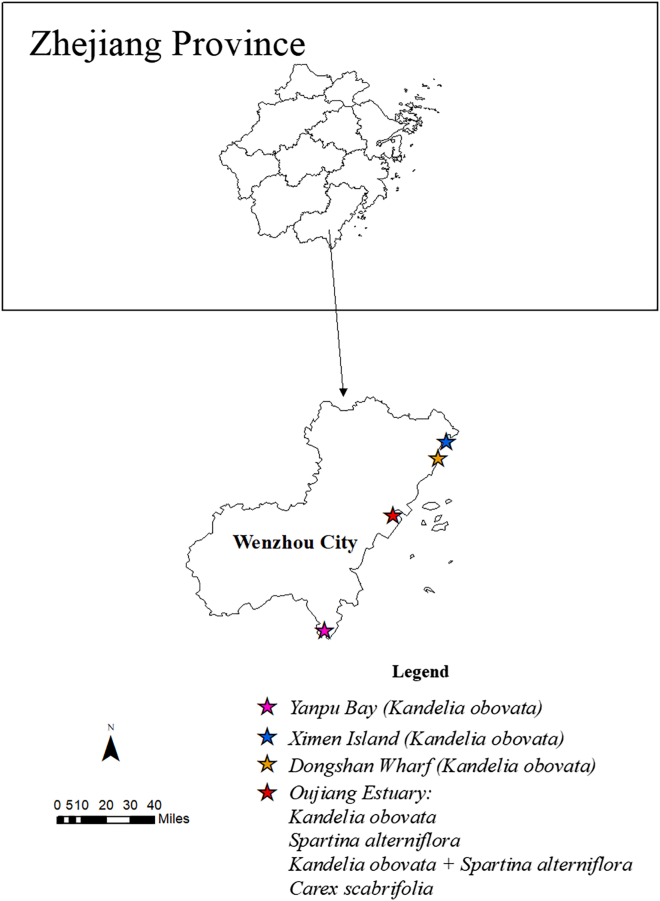
Map of the four vegetation types in Oujiang Estuary and four *Kandelia obovata* sites in Wenzhou City, Zhejiang Province

The present research project endeavors to offer a comprehensive exploration of the contrasting assessments of soil properties across different vegetation types (i.e., native *Carex scabrifolia*, invasive *S*. *alterniflora*, restored *K*. *obovata* forests, and mixed vegetation type of *K*. *obovata* and *S*. *alterniflora*) as well as across different *K*. *obovata* restoration sites, with sampling conducted throughout the 0–100 cm soil depth. These four distinct vegetation type zones were investigated in the Oujiang Estuary, and the restored *K*. *obovata* stands were sampled at four different locations (Dongshan Wharf, Oujiang Estuary, Ximen Island, and Yanpu Bay) in Zhejiang Province, China (Figure 1). Soil samples were collected and analyzed at depth intervals of 0–10, 10–20, 20–30, 30–40, 40–50, 50–70, and 70–100 cm. We hypothesized that (1) soil properties within the Oujiang Estuary vary with vegetation type and soil depth; (2) *K*. *obovata* forests improve soil quality compared with areas invaded by *S*. *alterniflora*; (3) *K*. *obovata* restoration alters the soil nutrient limitation regime of wetland ecosystems; and (4) soil properties in the restored *K*. *obovata* stands are site-specific.

## Results

### Variation in soil properties among vegetation types at the same soil depth

**Figure 2:**
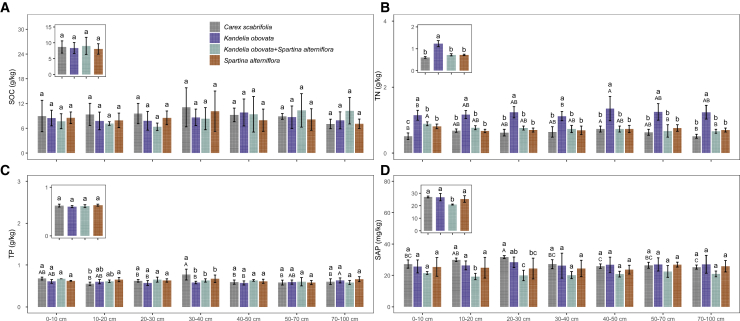
The distribution patterns of soil nutrients across vegetation types at different soil depths (A) SOC, (B)TN, (C) TP, and (D) SAP. SOC indicates soil organic carbon; TN indicates total nitrogen; TP indicates total phosphorus; SAP indicates soil available phosphorus. The top-left inset of each panel indicated the distribution patterns of soil nutrients among vegetation types throughout the 0-100 cm soil depth. Differences in uppercase letters signify significant differences among different soil depths within the identical vegetation type (*P* < 0.05). Differences in lowercase letters signify significant differences among different vegetation types at a given soil depth (*P* < 0.05). Data are presented as mean ± SEM (standard error of the mean)

The attributes of soil nutrients across the distinct vegetation zones at varying soil depths are depicted in Figure 2.

Across the 0–100 cm soil stratum, soil organic carbon (SOC) (8.07–9.04 g C kg^−1^) (Figure 2A) and total phosphorus (TP) concentration (0.60–0.63 g P kg^−1^) (Figure 2C) did not differ significantly among different vegetation types. The total nitrogen (TN) content under the *K*. *obovata* forest (1.23 ± 0.14 g N kg^−1^) was significantly higher than under mixed (0.72 ± 0.05 g N kg^−1^), *S*. *alterniflora* (0.72 ± 0.03 g N kg^−1^), and *C*. *scabrifolia* zones (0.60 ± 0.04 g N kg^−1^) (*p* < 0.05) (Figure 2B). The soil available phosphorus (SAP) content under the mixed vegetation type zones (20.92 ± 0.44 mg P kg^−1^) was significantly lower than that under the *C*. *scabrifolia* (27.05 ± 0.70 mg P kg^−1^), *K*. *obovata* (26.85 ± 2.92 mg P kg^−1^), and *S*. *alterniflora* zones (25.39 ± 2.63 mg P kg^−1^) (*p* < 0.05) (Figure 2D).

No significant differences in SOC content were observed among different vegetation types at any soil depth (Figure 2A). The *K*. *obovata* stands exhibited the highest TN content throughout the soil profile and was significantly higher than the *S*. *alterniflora*, *K*. *obovata* + *S*. *alterniflora*, and *C*. *scabrifolia* zones (*p* < 0.05) (Figure 2B). In contrast, the *C*. *scabrifolia* zone showed the lowest TN content across varying soil depths and was significantly lower than the other vegetation types at the surface soil depth (0–10 cm) (*p* < 0.05). For TP content, the *S*. *alterniflora* zone was significantly higher than the *C*. *scabrifolia* zone at the soil depth of 10–20 cm, whereas the latter showed significantly higher TP content than the other three vegetation types at the soil depth of 30–40 cm (Figure 2C) (*p* < 0.05). The mixed vegetation type zones displayed the lowest SAP content across soil depths, with significant differences at the soil depths of 10–20 cm and 20–30 cm (Figure 2D).

**Figure 3:**
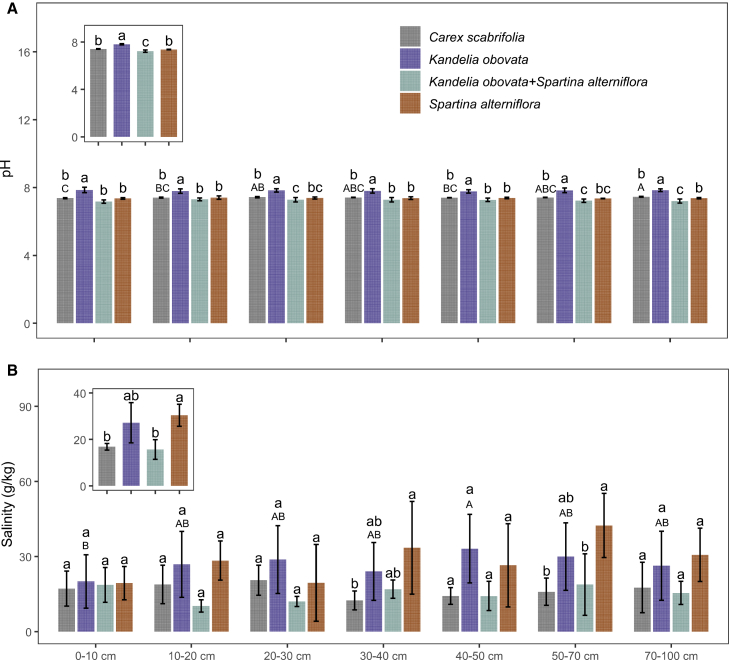
The distribution patterns of soil pH and salinity across vegetation types at different soil depths (A) soil pH and (B) soil salinity. The top-left inset of each panel indicates the distribution patterns of soil pH and salinity among vegetation types throughout the 0-100 cm soil depth. Differences in uppercase letters signify significant differences across soil depths within the identical vegetation type (*P* < 0.05). Differences in lowercase letters signify significant differences among different vegetation types at a given soil depth (*P* < 0.05). Data are presented as mean ± SEM (standard error of the mean).

Across the 0–100 cm soil profile, the highest pH value was observed in *K*. *obovata* forests (7.82 ± 0.05), which was significantly higher than that of the other three vegetation types (Figure 3A). Both *C*. *scabrifolia* (7.42 ± 0.02) and *S*. *alterniflora* (7.37 ± 0.03) also had significantly higher pH values than the mixed vegetation type (7.23 ± 0.10) (*p* < 0.05). Salinity under *S*. *alterniflora* zones (30.38 ± 4.77 g kg^−1^) was significantly higher than *C*. *scabrifolia* (16.81 ± 1.43 g kg^−1^) and *K*. *obovata* + *S*. *alterniflora* zones (15.61 ± 4.26 g kg^−1^) (*p* < 0.05) (Figure 3B).

The attributes of soil salinity and soil pH across various vegetation zones at differing soil depths are depicted in Figure 3. In comparison with the other vegetation type zones, the *K*. *obovata* forests exhibited significantly higher pH levels at different soil depths (*p* < 0.05) (Figure 3A). The mixed zone showed the lowest pH values, significantly lower than *C*. *scabrifolia* zones at the soil depth of 20–30, 50–70, and 70–100 cm. For soil salinity (Figure 3B), the *S*. *alterniflora* zone was significantly higher than *C*. *scabrifolia* at the soil depths of 30–40 cm and 50–70 cm (*p* < 0.05).

### Vertical distribution patterns of soil properties within each vegetation type

The effects of soil properties varied by both soil depth and vegetation type (Figure 2). Although no significant differences in SOC content were observed across soil depths for all vegetation types, distinct vertical trends were found (Figure 2A). SOC content in *S*. *alterniflora*, *C*. *scabrifolia*, and the mixed vegetation type zones increased initially and then decreased with soil depth, whereas in *K*. *obovata* forests it tended to increase along the soil profile. TN content also showed vegetation-specific vertical patterns (Figure 2B). In *K*. *obovata* and *C*. *scabrifolia*, TN content increased to a peak at 40–50 cm and then decreased with soil depth; this peak value was significantly higher than surface (0–10 cm) values (*p* < 0.05). Conversely, the mixed vegetation type showed a decreasing tendency, with surface TN content (0–10 cm) significantly higher than at the deeper soil depths (50–70 and 70–100 cm). For TP content (Figure 2C), the *K*. *obovata* forest exhibited an increasing trend with soil depth, with the highest value occurring at 70–100 cm, which was significantly higher than at the 20–30, 30–40, and 40–50 cm. In *C*. *scabrifolia* zones, TP content increased initially and then decreased, peaking at 30–40 cm, which was significantly higher than all other soil depths except 0–10 cm. Regarding the SAP content (Figure 2D), *C*. *scabrifolia* zones showed an initial increase followed by a decrease, with the peak value at 20–30 cm depth being significantly higher than that at all other soil depths except 10–20 cm. No significant differences in SAP content across soil depths were detected in *K*. *obovata*, *S*. *alterniflora*, or the mixed vegetation type.

Soil pH in the *C*. *scabrifolia* zones increased significantly with soil depth, reaching a maximum at 70–100 cm (Figure 3A), significantly higher than at 0–10, 10–20, and 40–50 cm depths (*p* < 0.05). In the *K*. *obovata* forest, soil salinity reached its highest level at 40–50 cm, which was significantly higher than that at the surface layer (0–10 cm) (Figure 3B).

### Site-scale variation of soil properties in *K*. *obavota* forests at the same soil depth

Throughout the 0–100 cm soil stratum, SOC (8.34–10.05 g C kg^−1^) and TN content (1.05–1.23 g N kg^−1^) did not differ significantly among sites. TP content varied significantly, being highest at Ximen Island (0.91 ± 0.04 g P kg^−1^) and significantly higher than that of the other three sites. TP contents at Dongshan Wharf (0.68 ± 0.04 g P kg^−1^) and Yanpu Bay (0.67 ± 0.03 g P kg^−1^) were also significantly higher than those at Oujiang Estuary (0.60 ± 0.02 g P kg^−1^). SAP was lowest at Yanpu Bay (22.95 ± 2.48 mg P kg^−1^), significantly lower than the other three sites (25.85–27.85 mg P kg^−1^).

**Figure 4:**
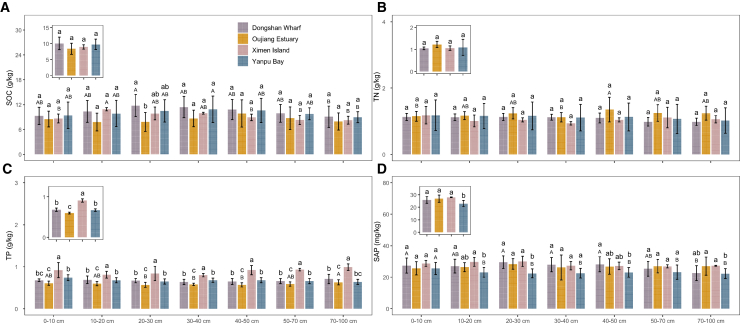
The distribution patterns of soil nutrients in *K. obovata* forests across sites at different soil depths (A) SOC, (B)TN, (C) TP, and (D) SAP. SOC indicates soil organic carbon; TN indicates total nitrogen; TP indicates total phosphorus; SAP indicates soil available phosphorus. The top-left inset of each panel indicated the distribution patterns of soil nutrients among different sites throughout the 0-100 cm soil depth. Differences in uppercase letters signify significant differences among different soil depths within the same site (*P* < 0.05). Differences in lowercase letters signify significant differences among different sites at a given soil depth (*P* < 0.05). Data are presented as mean ± SEM (standard error of the mean).

SOC content was consistently higher at Dongshan Wharf and Yanpu Bay than at Oujiang Estuary and Ximen Island across all soil depths (Figure 4A). A significant difference was observed at 20–30 cm depth, where Dongshan Wharf was significantly higher than the Oujiang Estuary. TN content did not differ significantly among sites were detected at any soil depth, although the highest TN content was consistently recorded at Oujiang Estuary (Figure 4B). Regarding TP content, Ximen Island and Oujiang Estuary exhibited the highest and the lowest TP content across the soil profile, respectively (Figure 4C). The TP content at Ximen Island was significantly higher than at the other three sites across different soil depths. Yanpu Bay displayed the lowest SAP content across the soil profile, with significant differences observed at the soil depths of 10–20 cm, 20–30 cm, 40–50 cm, and 70–100 cm (Figure 4D).

Throughout the 0–100 cm soil profile, soil pH differed significantly among sites (*p* < 0.05), ranking as follows: Dongshan Wharf (8.03 ± 0.05) > Yanpu Bay (7.95 ± 0.07) > Oujiang Estuary (7.82 ± 0.05) > Ximen Island (7.69 ± 0.02). For salinity, Ximen Island (42.26 ± 4.20 g kg^−1^) exhibited the highest value, which was significantly higher than those at the other three sites. Salinity at Oujiang Estuary (27.17 ± 8.67 g kg^−1^) and Yanpu Bay (32.57 ± 5.28 g kg^−1^) was also significantly higher than at Dongshan Wharf (10.98 ± 4.88 g kg^−1^).

**Figure 5:**
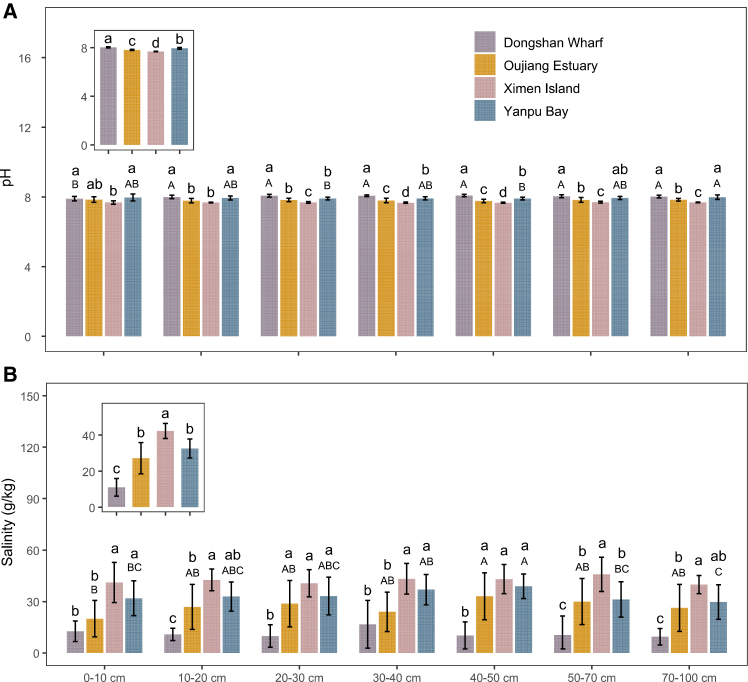
The distribution patterns of soil pH and salinity in *K. obovata* forests across sites at different soil depths (A) soil pH and (B) soil salinity. The top-left inset of each panel indicates the distribution patterns of soil pH and salinity among different sites throughout the 0-100 cm soil depth. Differences in uppercase letters signify significant differences across soil depths within the same site (*P* < 0.05). Differences in lowercase letters signify significant differences among different sites at a given soil depth (*P* < 0.05). Data are presented as mean ± SEM (standard error of the mean).

Among the four *K*. *obovata* restoration sites, soil pH varied significantly across depths (Figure 5A). Dongshan Wharf exhibited the highest soil pH values across soil depths, while Ximen Island showed the lowest; these differences were significant at all soil layers (*p* < 0.05). Additionally, the soil pH value at Yanpu Bay was significantly higher than that of Ximen Island across different soil layers (*p* < 0.05). A similar spatial pattern was observed for soil salinity, with Ximen Island displaying the highest values and Dongshan Wharf displaying the lowest at different soil layers; these differences were also significant at all soil depths (*p* < 0.05).

### Vertical distribution of soil properties within the same *K*. *obavota* forest sites

SOC content in *K*. *obovata* forests exhibited a consistent pattern of initial increase followed by a decrease with soil depth across all sites, though the magnitude of this trend differed significantly among Dongshan Wharf, Yanpu Bay, and Ximen Island. At Dongshan Wharf, TN generally decreased with depth, with significantly higher values in the upper soil layer (0–50 cm) than in the deeper layer (50–100 cm). In contrast, at Oujiang Estuary, the TN peaked in the middle soil layer (40–50 cm), where it was significantly higher than in the shallow soil layer (0–10 cm). No significant differences in TN content were found at Yanpu Bay or Ximen Island. TP and SAP content also showed distinct depth-related patterns. At Oujiang Estuary, TP content was significantly higher in the deepest soil layer (70–100 cm) than in the middle soil layer (20–50 cm). At Yanpu Bay, both TP and SAP content were highest in the shallow soil depth (0–10 cm), significantly exceeding those in deeper soil depths. At Dongshan Wharf, the SAP content was lowest at 70–100 cm and significantly lower than in the 0–10, 20–30, 30–40, and 40–50 cm soil layers.

Soil pH at Dongshan Wharf was significantly lower in the surface layer (0–10 cm) than in deeper soil layers. At Yanpu Bay, the soil pH was highest in the deepest soil layer (70–100 cm), which was significantly higher than that in the 20–30 cm and 40–50 cm soil layers. Soil salinity showed a consistent vertical pattern, characterized by an initial increase followed by a decrease with soil depth. The magnitude of this variation was particularly significant at the Oujiang Estuary and Yanpu Bay.

### Distribution of soil stoichiometric ratios among vegetation types at the same soil depth

**Figure 6:**
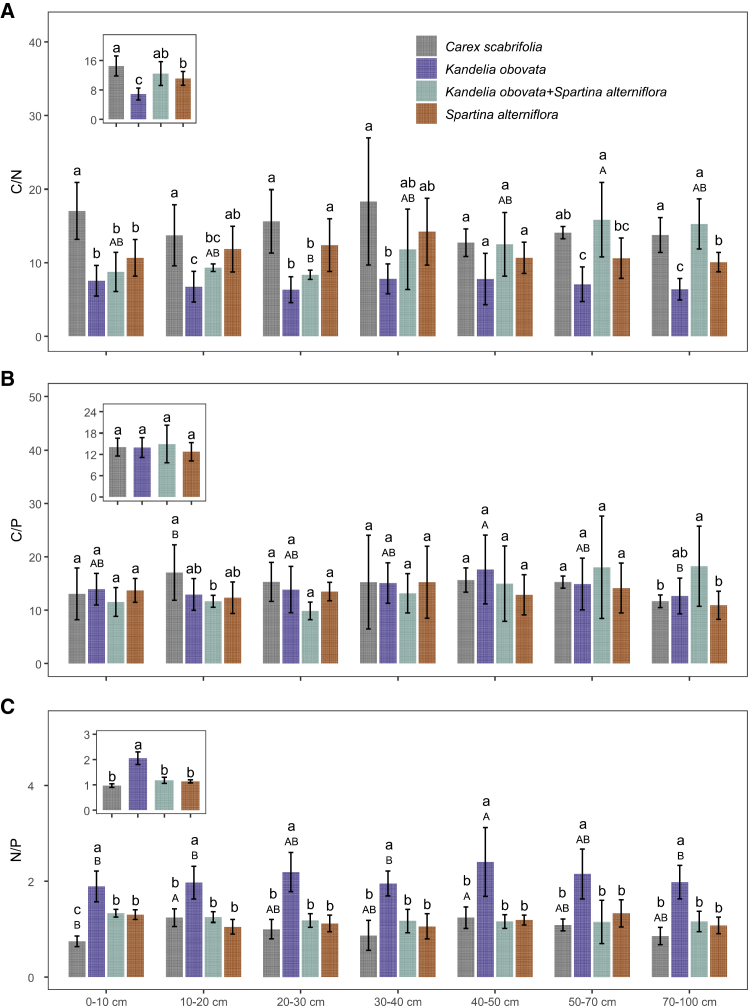
The distribution patterns of soil stoichiometric ratios across different vegetation types at different soil depths (A) C/N, (B) C/P, and (C) N/P. The top-left inset of each panel indicates the distribution patterns of soil C/N, C/P, and N/P ratios among vegetation types throughout the 0-100 cm soil depth. Different uppercase letters represent significant differences among different soil depths under the same vegetation type (*P* < 0.05). Different lowercase letters represent significant differences among different vegetation types at the same soil depth (*P* < 0.05). Data are presented as mean ± SEM (standard error of the mean).

Soil stoichiometric ratios (C:N, C:P, and N:P) among vegetation types and across different soil depths are shown in Figure 6. Throughout the soil stratum spanning from 0 to 100 cm in depth, the C:N ratio in the *C*. *scabrifolia* zone (14.49 ± 2.71) was significantly higher than that in the *K*. *obovata* (6.90 ± 1.62) and *S*. *alterniflora* zones (11.11 ± 1.88) (*p* < 0.05). However, no significant differences in the C:P ratio were observed among vegetation types. For the N:P ratio, the *K*. *obovata* forest (2.05 ± 0.25) exhibited significantly higher values than the *C*. *scabrifolia* zone (0.97 ± 0.07), *S*. *alterniflora* (1.14 ± 0.06), and mixed vegetation type (1.18 ± 0.12) (*p* < 0.05).

In the upper soil profile (0–50 cm), the *C*. *scabrifolia* zone showed the highest C:N ratio, being significantly higher than in the *K*. *obovata* zone throughout 0–40 cm and higher than the mixed vegetation type within 10–30 cm (Figure 6A). In deeper soil layers (50–70 cm and 70–100 cm), the mixed vegetation type zone exhibited the highest C:N ratio, significantly exceeding those in the *K*. *obovata* and *S*. *alterniflora* zones. The mixed vegetation type had the lowest C:P ratio in the 0–40 cm depth, significantly lower than in the *C*. *scabrifolia* zone at 10–20 cm. In contrast, in the 40–100 cm layer, the *S*. *alterniflora* zones showed the lowest C:P ratio, significantly below that of the mixed vegetation type at 70–100 cm. Notably, *K*. *obovata* maintained the highest N:P ratio across all soil depths, significantly higher than the other three vegetation types.

### Distribution of soil stoichiometric ratios across different soil depths within the same vegetation type

The C:N ratio showed significant disparities with soil depths in the *C*. *scabrifolia* zone (*p* < 0.05), with the value in the 50–70 cm layer being significantly higher than that in the 20–30 cm layer. For the C:P ratio, significant depth-related differences were identified in the *K*. *obovata* regions (*p* < 0.05), where the ratio in the 40–50 cm layer was significantly higher than that in the 10–20 and 70–100 cm layers. As for the N:P ratio, the notable disparities with depth were found in both the *K*. *obovata* and *C*. *scabrifolia* zones (*p* < 0.05). In both vegetation types, the N:P ratio exhibited a unimodal pattern, initially increasing and then decreasing with soil depths. Specifically, in the *K*. *obovata* zones, the N:P ratio at 40–50 cm was significantly higher than that at 0–20, 30–40, and 70–100 cm. In the *C*. *scabrifolia* zones, the N:P ratios at 10–20 and 40–50 cm were significantly higher than those at 0–10 cm.

### Distribution of soil stoichiometric ratios among different sites at the same soil depth

In the 0–100 soil stratum, there were no significant differences in the C:N ratio across sites. However, the C:P ratio at Ximen Island was significantly lower than the other three sites. Oujiang Estuary exhibited the highest N:P ratio and was significantly higher than Dongshan Wharf and Ximen Island.

**Figure 7:**
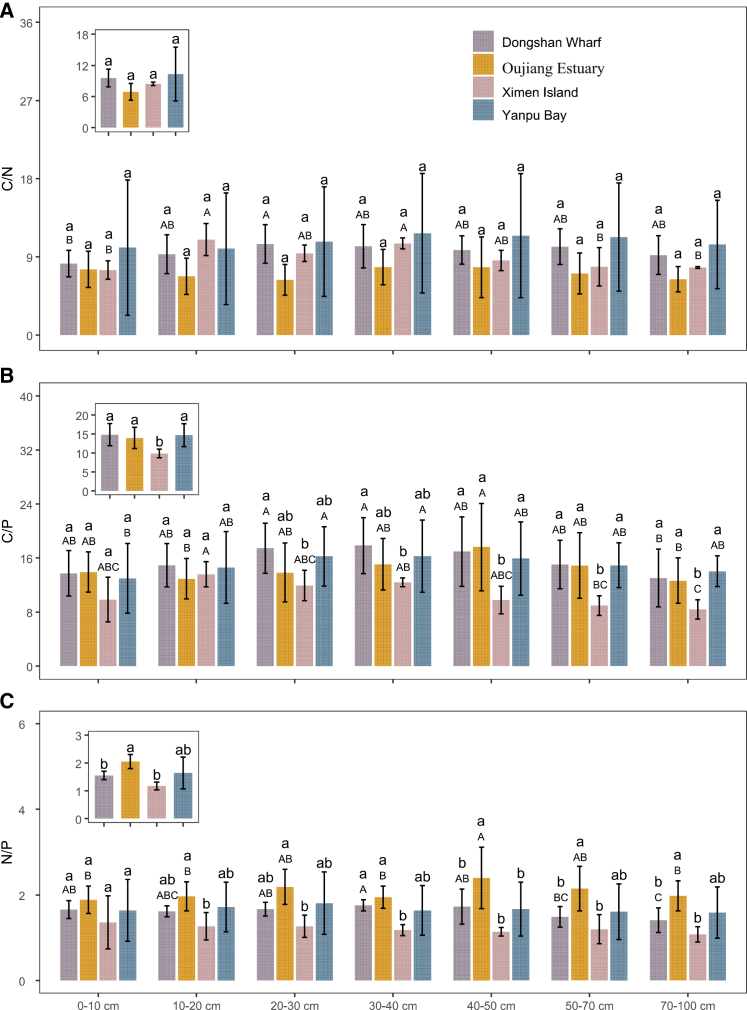
The distribution patterns of soil stoichiometric ratios in *K. obovata* forests across different sites at different soil depths (A) C/N, (B) C/P, and (C) N/P. The top-left inset of each panel indicates the distribution patterns of soil C/N, C/P, and N/P ratios among different sites throughout the 0-100 cm soil depth. Different uppercase letters represent significant differences among different soil depths at the same site (*P* < 0.05). Different lowercase letters represent significant differences among different sites at the same soil depth (*P* < 0.05). Data are presented as mean ± SEM (standard error of the mean).

The C:N ratio demonstrated no significant differences among the four study sites at the same soil depth. In contrast, both the C:P and N:O ratios exhibited marked spatial variations. The C:P ratio was consistently lowest at Ximen Island, showing statistically significant reductions compared to Dongshan Wharf (within the 20–100 cm soil layer) and Oujiang Estuary (within the 40–100 cm soil layer). Concurrently, the N:P ratio was also minimal at Ximen Island throughout the sampled depth and significantly lower than that at Oujiang Estuary in the 10–100 cm layer (Figure 7).

### Distribution of soil stoichiometric ratios across soil depths within each site

The C:N, C:P, and N:P ratios across different sites displayed a consistent trend of initial increase followed by a decrease with soil depth. Specifically, at Dongshan Wharf, the C:N ratio in the 20–30 cm soil depth was significantly higher than that in the 0–10 cm soil depth. At Ximen Island, the C:N ratios in the soil depth of 10–20 and 30–40 were significantly higher than those in the soil layer of 0–10, 50–70, and 70–100 cm layers.

For the C:P ratio, the values in the 20–30 and 30–40 cm at Dongshan Wharf were significantly higher than those in the 70–100 cm. At Oujiang Estuary, the C:P ratio in the 40–50 cm layer was significantly higher than in the 10–20 and 70–100 cm layers. At Yanpu Bay, C:P values in the 20–30 and 30–40 cm layers were significantly higher than in the first layer (0–10 cm). At Ximen Island, the C:P ratio in the 10–20 cm layer was significantly higher than that in the 50–70 and 70–100 cm layers.

For the N:P ratio, values in the 30–40 cm layer at Dongshan Wharf were significantly higher than those in the 50–70 and 70–100 cm layers. At Oujiang Estuary, the N:P ratio in the 40–50 cm layers was significantly higher than in 0–10, 10–20, 30–40, and 70–100 cm layers.

### Correlation analysis

**Figure 8:**
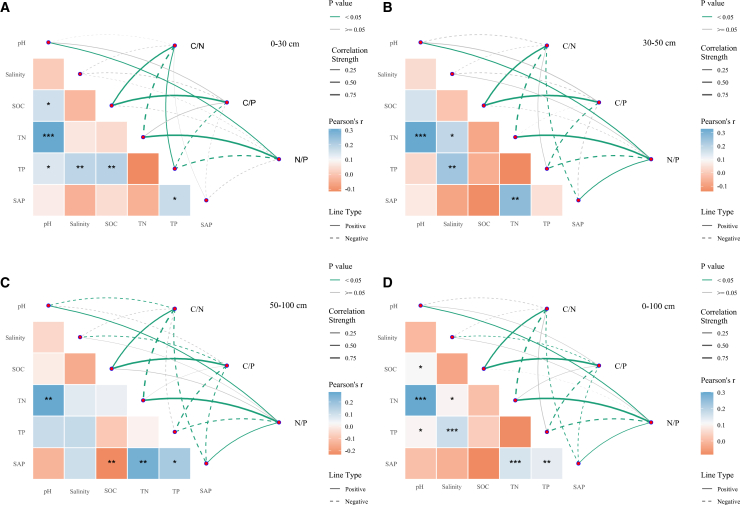
Results of Mantel tests illustrating the relationships between soil stoichiometric ratios (C:N, C:P, and N:P) and soil properties at various soil layer intervals (A) 0–30, (B) 30–50, (C) 50–100, and (D) 0–100 cm. Note: SOC refers to soil organic carbon; TN refers to total nitrogen; TP refers to total phosphorus; SAP refers to soil available phosphorus.

Soil pH exhibited depth-dependent correlations with soil nutrients: positively with SOC, TN, and TP at 0–30 cm and 0–100 cm soil depths but only with TN at deeper soil depths (30–50 cm and 50–100 cm). Soil salinity correlated positively with TP at 0–30 cm, 30–50 cm, and 0–100 cm depths, and with TN at 30–50 cm and 0–100 cm. SOC correlated positively with TP at 0–30 cm, but negatively with SAP at 50–100 cm. Both TN and TP showed consistent positive correlations with SAP across most depth intervals (Figure 8).

Correlations between stoichiometric ratios (C:N, C:P, and N:P) and soil properties also varied significantly with soil depth (Figure 8). The C:N ratio correlated positively (*p* < 0.05) with soil pH, TN, SOC, and SAP at soil depths of 0–30 cm, 50–100 cm, and across the entire 0–100 cm profile but only with TN and SOC at 30–50 cm. The C:P ratio correlated with multiple variables depending on depth: with TN, TP, SOC, SAP, and soil salinity at 0–30 cm (*p* < 0.05); with TP and SOC at 30–50 cm; and with soil pH, TP, SOC, and SAP at 50–100 cm (*p* < 0.05). Across the full profile (0–100 cm), the C:P ratio was linked to TP, SOC, and SAP. Conversely, the N:P ratio maintained a consistent positive correlation with soil pH, TN, and TP across all examined soil depths (*p* < 0.05).

### Principal-component analysis based on soil properties for different vegetation types

At the examined soil depths (0–30 cm, 30–50 cm, 50–100 cm, and 0–100 cm), the eigenvalues of the first three principal components (PC1, PC2, and PC3) all exceeded 1 and together explained 79.57%–82.55% of the total variance (Table 1), effectively capturing soil-property variability and overall soil condition.

**Table 1:** Initial eigenvalues of principal-component analysis of soil properties across different vegetation types

Soil depth	principal component	initial eigenvalues		
		eigenvalue	contribution rate/%	cumulative contribution rate/%
0–30 cm	PC1	3.677	40.85	40.85
	PC2	2.449	27.21	68.06
	PC3	1.183	13.14	81.20
30–50 cm	PC1	3.816	42.40	42.40
	PC2	2.365	26.28	68.68
	PC3	1.232	13.68	82.36
50–100 cm	PC1	3.775	41.94	41.94
	PC2	2.520	28.00	69.94
	PC3	1.134	12.60	82.55
0–100 cm	PC1	3.559	39.54	39.54
	PC2	2.350	26.11	65.65
	PC3	1.253	13.92	79.57

Based on the principal-component analysis (PCA), the comprehensive evaluation of wetland soil properties was conducted using the integrative score derived from the contribution rates of each principal component (Table 2). Standardized data were substituted into the integrative score equation, with higher integrative scores indicating better soil quality (Table 3). *K*. *obovata* consistently showed the highest score across all soil depths, while the mixed vegetation type ranked lowest at 0–30 cm and *C*. *scabrifolia* lowest at deeper intervals.

**Table 2:** The integrative score formula for different vegetation types and *K*. *obovata* at different soil depths

Type	soil depth	integrative score formula
Four vegetation types	0–30 cm	F = 0.409 F1 + 0.272 F2 + 0.131 F3
	30–50 cm	F = 0.424 F1 + 0.263 F2 + 0.137 F3
	50–100 cm	F = 0.419 F1 + 0.280 F2 + 0.126 F3
	0–100 cm	F = 0.395 F1 + 0.261 F2 + 0.139 F3
*K*. *obovata* forest in different sites	0–30 cm	F = 0.318 F1 + 0.246 F2 + 0.149 F3 + 0.119 F4
	30–50 cm	F = 0.350 F1 + 0.248 F2 + 0.156 F3
	50–100 cm	F = 0.330 F1 + 0.259 F2 + 0.147 F3
	0–100 cm	F = 0.320 F1 + 0.248 F2 + 0.149 F3 + 0.115 F4

**Table 3:** Principal components, composite scores, and ranking of soil properties in different vegetation types

Soil depth	vegetation types	score			integrative score	ranking
		the primary principal component (F1)	the secondary principal component (F2)	the third principal component (F3)		
0-30 cm	*Carex scabrifolia*	−1.0263	1.0017	1.5065	0.0513	3
	*Kandelia obovata*	−0.2887	0.3187	1.5900	0.1777	1
	*Spartina alterniflora*	−0.7022	0.3126	0.9230	0.1065	2
	*Kandelia obovata* + *Spartina alterniflora*	−0.6220	0.2699	1.2262	−0.0195	4
30-50 cm	*Carex scabrifolia*	−0.5394	0.5674	−0.8907	−0.2015	4
	*Kandelia obovata*	0.2940	0.1674	−1.2390	−0.0008	1
	*Spartina alterniflora*	−0.4134	0.6261	−0.8087	−0.0231	2
	*Kandelia obovata* + *Spartina alterniflora*	−0.0324	0.1299	−1.1347	−0.1348	3
50-100 cm	*Carex scabrifolia*	−0.6510	−0.7485	0.1457	−0.4642	4
	*Kandelia obovata*	0.1209	−0.7137	0.6271	−0.0701	1
	*Spartina alterniflora*	−1.2201	−0.0549	0.0540	−0.2249	3
	*Kandelia obovata* + *Spartina alterniflora*	0.0621	−0.9299	0.9831	−0.1105	2
0-100 cm	*Carex scabrifolia*	−0.7071	0.1146	0.9809	−0.1131	4
	*Kandelia obovata*	0.0197	−0.2146	1.0458	0.0974	1
	*Spartina alterniflora*	−0.7557	−0.0373	0.8227	0.0768	2
	*Kandelia obovata* + *Spartina alterniflora*	−0.2133	−0.0511	1.0067	0.0424	3

### PCA based on soil properties for *K*. *obovata* forests across different sites

At 0–30 cm and 0–100 cm, four principal components (PC1, PC2, PC3, and PC4) had eigenvalues >1, together explaining >83% of variance (Table 4). At 30–50 cm and 50–100 cm, three components (PC1, PC2, and PC3) explained >73% of variance, sufficiently representing soil-property variation.

**Table 4:** Initial eigenvalues of principal-component analysis of soil properties in different sites

Soil depth	principal component	initial eigenvalues		
		eigenvalue	contribution rate/%	cumulative contribution rate/%
0-30 cm	PC1	2.8605	0.3178	0.3178
	PC2	2.2136	0.2460	0.5638
	PC3	1.3361	0.1485	0.7123
	PC4	1.0697	0.1189	0.8311
30-50 cm	PC1	3.1484	0.3498	0.3498
	PC2	2.2345	0.2483	0.5981
	PC3	1.4005	0.1556	0.7537
50-100 cm	PC1	2.9720	0.3302	0.3302
	PC2	2.3318	0.2591	0.5893
	PC3	1.3244	0.1472	0.7365
0-100 cm	PC1	2.8761	0.3196	0.3196
	PC2	2.2342	0.2482	0.5678
	PC3	1.3372	0.1486	0.7164
	PC4	1.0384	0.1154	0.8318

Standardized data were substituted into the integrative score equation, with the resulting scores across soil depths summarized in Table 5. The rankings indicate that Ximen Island and Yanpu Bay consistently outperformed the other two sites, whereas Dongshan Wharf exhibited the lowest score throughout the soil depths.

**Table 5:** Principal components, composite scores, and ranking of soil properties of *K*. *obovata* in different sites

Soil depth	site	score				integrative score	ranking
		the primary principal component (F1)	the secondary principal component (F2)	the third principal component (F3)	the Fourth principal component (F4)		
0–30 cm	Dongshan Wharf	0.9546	0.2138	−0.3762	−0.7694	0.2085	4
	Oujiang Estuary	0.4639	0.7834	0.7167	−0.2714	0.4143	3
	Ximen Island	0.4564	0.9430	1.3487	−0.1204	0.5630	2
	Yanpu Bay	0.6428	0.6655	1.2863	0.0472	0.5646	1
30–50 cm	Dongshan Wharf	−0.7844	−0.2112	−0.7749	–	−0.4474	4
	Oujiang Estuary	−0.3181	0.5066	0.3289	–	0.0657	3
	Ximen Island	−0.1048	1.0442	0.7841	–	0.3446	1
	Yanpu Bay	−0.5945	0.8702	0.9806	–	0.1607	2
50–100 cm	Dongshan Wharf	−0.5796	−0.3275	0.4192	–	−0.2146	4
	Oujiang Estuary	0.0206	−0.7500	1.2020	–	−0.0106	1
	Ximen Island	0.1361	−1.0940	1.2915	–	−0.0484	2
	Yanpu Bay	−0.3857	−0.7641	1.1702	–	−0.1531	3
0–100 cm	Dongshan Wharf	0.7300	0.1944	−0.2427	−0.9717	0.1334	4
	Oujiang Estuary	0.2294	0.6731	0.8442	−0.3603	0.3243	3
	Ximen Island	0.1293	0.9940	1.3175	−0.2294	0.4574	2
	Yanpu Bay	0.5425	0.6784	1.1936	0.0842	0.5288	1

## Discussion

The enrichment of nitrogen in the soil and the improved utilization of phosphorus facilitate the invasion of *S*. *alterniflora* in China’s eutrophic coastal areas.^6^ Nevertheless, the growing prevalence of such invasive plants in wetland regions may pose a threat to the ecosystem’s capability to store carbon, nitrogen, and phosphorus.^37^ Specifically, *S*. *alterniflora* invasion may potentially impair the ability of mangroves to store carbon and eliminate nitrogen.^38^^,^^39^ Consequently, these findings collectively suggest that the substitution of indigenous salt marsh species or mangrove species by *S*. *alterniflora* should not be regarded as a natural climate-related solution for increasing the carbon sinks in China’s coastal regions,^21^ but rather a serious environmental challenge.^37^ Conversely, replacing *S*. *alterniflora* with native salt marsh species or mangrove species should be considered a natural climate-related solution.

In this context, ecological restoration by using the cold-tolerant mangrove *K*. *obovata* has emerged as a promising strategy. It not only enhances the quality of degraded coastal wetlands^25^ but also effectively curbs the spread of *S*. *alterniflora* in China.^34^^,^^40^ The northward introduction of mangroves to replace *S*. *alterniflora* is thus regarded as a highly efficacious ecological strategy for coastal restoration,^34^ with well-developed mature afforested mangroves demonstrating a remarkable carbon storage capacity. Consequently, it is more advisable to carry out the mangrove restoration with the eradication of *S*. *alterniflora* in subtropical regions.^32^ Both the invasion of *S*. *alterniflora* and the rehabilitation of *K*. *obovata* alter soil properties,^29^^,^^40^ which in turn mediate ecosystem nutrient cycling. The extent of carbon sequestration capacity of the introduced mangroves is also affected by a range of site-specific factors, such as climate characteristics, edaphic properties, and land-use history.^41^ To elucidate these dynamics, this study employs an integrative design, encompassing four vegetation types and four mangrove restoration sites, with sampling conducted throughout the 0–100 cm soil depth. This systematic approach yields more holistic and practical insights for ecosystem restoration, whereas previous studies have often been constrained by a narrower focus on single vegetation types, individual restoration sites, or surface soil layers.

### SOC

A multitude of findings have shown that mangrove restoration can effectively enhance SOC storage,^39^^,^^42^^,^^43^ with vegetation type being a key influencing factor.^43^^,^^44^ However, the trajectory of SOC accumulation is time dependent. The newly restored stands^6^^,^^45^ may not increase the SOC content compared to invasive *S*. *alterniflora* zones. In contrast, fully developed and mature mangroves show significantly higher SOC levels than *S*. *alterniflora* across surface and deeper soil layers.^9^^,^^33^^,^^34^^,^^41^^,^^45^^,^^46^ This difference can be ascribed to the ecological stability and well-established restoration effects demonstrated by the mature mangroves.^31^ Consistent with this early-stage pattern, the restored *K*. *obovata* stands in this study (aged 6–7 years) did not exhibit a higher SOC content than the other three vegetation types (*S*. *alterniflora*, *Carex scabrifolia*, and mixed stands) across nearly 0–100 cm soil depths. Furthermore, no significant differences in SOC concentration were detected among the four restored *K*. *obovata* sites. These results align with findings that restored mangroves often show similar SOC levels with *S*. *alterniflora* but lower levels than the natural forests,^47^^,^^48^ underscoring that the carbon storage functions of restored mangroves may require extended time or targeted management to fully develop.^48^ The influence of the invasion of *S*. *alterniflora* on SOC content also appears to be time-sensitive. Certain research revealed that in invaded wetlands, the SOC content first showed an increase, but this was only on a decadal scale, and subsequently decreased.^21^ Moreover, ecosystem context appears to modulate these effects. In a mangrove ecosystem dominated by *K*. *obovata*, the invasion of *S*. *alterniflora* led to a reduction in the SOC content within the upper 60 cm soil layer.^21^ However, no substantial discrepancies in the SOC content between the sites restored with *K*. *obovata* and those invaded by *S*. *alterniflora*,^17^ which aligns with the results of the present research. Collectively, the lack of SOC differentiation in our study likely reflects the relatively young age of the restored *K*. *obovata* stands. This highlights the importance of considering temporal dynamics when evaluating the carbon sequestration potential of mangrove restoration projects, as detectable SOC gains may emerge only after longer periods of ecosystem development.

### TN, TP, and SAP

Exotic plants are capable of surmounting nitrogen limitation by enhancing soil nitrogen availability,^49^^,^^50^ thereby guaranteeing successful invasion.^51^ In the current research, the TN content in the zones of *K*. *obovata* forests was notably greater than in the zones dominated by *C*. *scabrifolia*, *S*. *alterniflora*, and the mixed vegetation type throughout the 0–100 cm soil layer. This discovery aligns with earlier reports indicating that mangroves generally maintain higher TN content compared to *S*. *alterniflora*-invaded regions.^17^^,^^52^ The lower TN content in *S*. *alterniflora*-invaded regions was mainly due to the lower nitrogen mineralization and immobilization rates in the soils.^49^^,^^50^ The effect of mangrove restoration on TN content also appears to be time dependent: short-term mangrove restoration may initially reduce TN content, whereas long-term restoration tends to increase it,^9^ with mature mangroves exhibiting the highest TN levels.^6^ Moreover, no significant differences were found in TN content among various restored sites in this study. Collectively, these patterns suggest that nitrogen availability is likely not a limiting factor in the restored mangrove forests compared with the invaded *S*. *alterniflora* and native salt marsh species.

The effects of *S*. *alterniflora* invasion on soil TP or SAP content remain inconsistent across studies, with reports indicating either a substantial increase^6^ or decrease.^53^ Mangrove restoration, on the other hand, appears to exert a minor influence on TP content.^6^ Consistent with earlier findings,^52^ no significant disparities in TP content were detected between areas invaded by *S*. *alterniflora* and those where mangroves were restored. The relatively stable TP levels in wetlands under different vegetation types may reflect the inherent conservation of phosphorus cycling, which involves minimal atmospheric exchange and is more resistant to perturbations caused by degradation or restoration processes.^54^ Conversely, the observed spatial variability in SAP among different restored *K*. *obovata* sites indicates that the phosphorus availability may act as a limiting factor in wetland nutrient cycling.^55^ The lowest soil SAP content recorded in the mixed vegetation type zones indicates that a considerable amount of phosphorus was either utilized or translocated as a result of the growth of the vegetation species. Functioning as a biological pump, vegetation moves phosphorus from the soil into plant tissue and then releases substantial amounts during inundation events. This phenomenon partially explains the observed decreases in TP and SAP content within these vegetation communities.

### C:N, C:P, and N:P ratios and soil quality

In this present study, soil C:N ratios ranged from 6.90 to 14.49 among different vegetation types and ranged from 6.90 to 10.34 among different restored *K*. *obovata* sites. These values are notably lower than those reported for other coastal wetlands (25.2)^56^ and mangrove ecosystems (ranging from 10.6 to 28.2).^57^^,^^58^^,^^59^ The relatively lower ratios of C:N observed here are likely ascribed to the quicker mineralization or breakdown of soil organic matter^60^ in conjunction with the relatively lower SOC content (8.38–9.04 g/kg among different vegetation types; 8.38–10.05 g/kg among different restored sites) when compared with the other coastal wetlands (20.00 g/kg)^56^ and mangrove ecosystems (ranging from 16 to 34 g/kg).^57^^,^^58^^,^^59^ Furthermore, compared with most wetlands (0.1 g/kg)^56^ and mangrove forests (0.2–0.8 g/kg),^57^^,^^58^^,^^59^ the TP content in the studied sites was higher (0.60–0.63 g/kg among different vegetation types; 0.60–0.91 g/kg among various restored sites). Nevertheless, both the C:P (12.75–14.19 across different vegetation types; 9.86–14.83 across different restored sites) and N:P ratios (0.97–2.05 among different vegetation types; 1.55–2.05 among different restored sites) were considerably lower than the reference value for most wetlands (C:P ≈ 202.00; N:P ≈ 8.0)^56^ and for mangroves in China (C:P: 27.8–136.2; N:P: 2.4–6.0).^57^^,^^58^^,^^59^ These phenomena can probably be attributed to the abundant phosphorus availability coupled with restricted carbon and nitrogen accumulation in the soil. Notably, the restored *K*. *obovata* forests exhibited the lowest soil C:N and highest N:P ratios among all vegetation types. Coupled with their significantly higher TN content, the lowest C:N ratio indicates a state of relatively high nitrogen availability within this system. Simultaneously, the highest N:P ratio, observed alongside non-significant differences in TP content, reveals a potential phosphorus limitation relative to nitrogen. These stoichiometric patterns collectively demonstrate that *K*. *obovata* restoration alters the soil nutrient regime toward a pattern characterized by nitrogen sufficiency coupled with probable phosphorus limitation. This altered nutrient status has important implications for understanding ecosystem nutrient cycling, organic matter decomposition, and long-term productivity in these restored mangroves. Critically, the shift toward phosphorus limitation may also suppress the growth of *S*. *alterniflora*, which typically thrives under nitrogen-replete conditions.^61^ Consequently, ecological replacement using *K*. *obovata* represents an effective approach for controlling *S*. *alterniflora* invasion.

This study characterized and assessed the soil properties across the 0–100 cm profile in coastal wetlands of Zhejiang Province, China. We compared the soil properties among different vegetation types in Oujiang Estuary (native salt marshes, invasive *S*. *alterniflora*, restored *K*. *obovate* forests, and the mixed *K*. *obovata* and *S*. *alterniflora* stands) and among multiple restored *K*. *obovata* sites (Dongshan Wharf, Oujiang Estuary, Ximen Island, and Yanpu Bay). The results demonstrated that soil properties were significantly influenced by vegetation type and soil depths, and restored *K*. *obovata* forests also exhibited site-specific variations. Among the vegetation types, *K*. *obovata* stands exhibited the highest TN and SAP content as well as the highest pH value, across the 0–100 cm profile. In contrast, *S*. *alterniflora* invasion was associated with the highest soil salinity. Mantel test indicated that soil stoichiometric ratios were closely linked to corresponding nutrient content and other key properties. The C:N ratio correlated negatively with SAP content, the C:P ratio negatively with salinity, and the N:P ratio positively with soil pH. The consistently low C:N, C:P, and N:P ratios across the study area suggest that nitrogen, rather than phosphorus, is the primary limiting nutrient in these coastal soils. Based on the comprehensive assessment of soil conditions, the restored *K*. *obovata* forests ranked the highest soil quality among vegetation types, and Ximen Island and Yanpu Bay showed the highest potential for restoration among sites. Overall, this work demonstrates that the northward afforestation with *K*. *obovata* to control *S*. *alterniflora* invasion can effectively enhance soil conditions and improve the carbon storage potential of coastal wetlands. However, this restoration approach also alters soil nutrient cycling and increases ecosystem demand for phosphorus. Therefore, to ensure the long-term health, stability, and carbon sequestration capacity of these restored mangrove ecosystems, future *K*. *obovata* restoration and management strategies should consider incorporating targeted phosphorus supplementation in Zhejiang Province, China.

### Limitations of the study

A limitation of this study is its reliance on a short-period sampling campaign to characterize soil properties to a depth of 0–100 cm. This approach does not capture the long-term dynamics of soil properties, which can vary with season, year, and mangrove stand age. Future long-term monitoring is therefore necessary to determine whether the observed trends, particularly regarding phosphorus limitation, persist or intensify over decadal timescales.

## Resource availability

### Lead contact

Further information and requests for resources and reagents should be directed to and will be fulfilled by the lead contact, Sheng Yang (yangs@zaas.ac.cn).

### Materials availability

This study did not generate new unique reagents.

### Data and code availability

• All data reported in this paper will be shared by the lead contact upon request.
• This study does not report original code.
• Any additional information required to reanalyze the data reported in this article is available from the lead contact upon request.

## Acknowledgments

This research was financially supported by the Key R&D Program of Zhejiang Province, China (2023C02003); Wenzhou High-level Innovation Team “Coastal Characteristic Plant Innovation and Utilization Project” (NY202401); and Open Project of the Engineering Research Center for Southeast Coastal Characteristic Plants of National Forestry and Grassland Administration (LCDNZX2025002).

## Author contributions

Y.Z., conceptualization, methodology, data curation, formal analysis, and writing – original draft; S.L., methodology and investigation; J.W., methodology and investigation; X.L., methodology and investigation; W.X., methodology and investigation; X.L., methodology and investigation; H.L., methodology and investigation; Q.C., data curation, formal analysis, writing – review and editing; S.Y., data curation, formal analysis, writing – review and editing; all authors have read and agreed to the published version of the manuscript.

## Declaration of interests

The authors declare no conflicts of interests.

## Declaration of generative AI and AI-assisted technologies in the writing process

During the preparation of this work, the authors did not use any generative AI or AI-assisted technologies that generate original content. All content is the original work of the authors, who take full responsibility for the accuracy.

## STAR★Methods

### Key resources table

**Table:** 

REAGENT or RESOURCE	SOURCE	IDENTIFIER
**Software and algorithms**		

R software (version 4.3.0)	The R Development Core Team^62^	https://cran.r-project.org/
RStudio	Posit Software, PBC^63^	https://posit.co/

### Experimental model and study participant details

Omitted as our study does not involve biological models.

### Method details

#### Study area

This research was carried out in the coastal areas of Wenzhou City, Zhejiang province, China. The climate in the study area is a mid-subtropical monsoon climate, characterized by a distinct seasonal alternation of winter and summer monsoons. The mean annual temperature spans from 17.3 to 19.4°C, with a mean temperature of 4.9∼9.9°C in January and 26.7∼29.6°C in July. The annual precipitation amounts to 1113∼2494 mm.

#### Experimental design, field sampling, and laboratory analysis

##### Comparison of four vegetation types in Oujiang Estuary

*K*. *obovata* was transplanted for ecological restoration in the Oujiang Estuary in 2018, where the exotic *S*. *alterniflora* had been removed. The dominant native salt marsh species in this study area is *C. scabrifolia*. Four distinct vegetation types were examined, each represented by a minimum of three replicate plots: *K*. *obovata* (12 plots), *C*. *scabrifolia* (3 plots), *S*. *alterniflora* (3 plots), and a mixed community of *K*. *obovata* and *S*. *alterniflora* (3 plots).

##### Comparison of *K*. *obovata* forests across four regions

To evaluate the site-specific effects of *K*. *obovata* restoration on soil properties across different geographical regions, four separate sites were selected: Dongshan Wharf, Oujiang Estuary, Ximen Island, and Yanpu Bay (Figure 1). A total of 45 plots were sampled: 9, 12, 3, and 21 plots at each site, respectively. All sampled plots were characterized by a *K*. *obovata*-dominated forest community. All *K*. *obovata* stands had been planted during 2017-2018.

##### Soil sampling and laboratory analysis

Field soil sampling was conducted throughout the growing season in 2023. In each plot, three soil cores were sampled per depth interval (i.e., 0-10 cm, 10-20 cm, 20-30 cm, 30-40 cm, 40-50 cm, 50-70 cm, and 70-100 cm) and combined into one composite sample. Prior to soil sampling, the litter layers were removed. The soil samples were preserved until laboratory processing for physicochemical property analysis.

The soil pH value^64^ and salinity^40^ were measured. The evaluation of SOC was carried out through dichromate oxidation combined with colorimetric determination.^65^ The quantification of soil TN content was performed via the Kjeldahl analytical technique.^66^ The measurement of soil TP content was conducted through a colorimetric method after digesting the samples with H_2_SO_4_ and HClO_4_.^67^ The quantification of SAP contents was achieved through the colorimetric method, utilizing a 0.5 M NaHCO_3_ solution.^68^

### Quantification and statistical analysis

#### Statistical analysis

Statistical analyses were performed employing the R software, version 4.5.1. Two-way analysis of variance (ANOVA) was implemented with the objectives of evaluating 1) the effects of vegetation type (within Oujiang Estuary) and soil depth, and 2) the influence of *K*. *obovata* restoration sites (across different regions) and soil depth on a range of soil properties and soil stoichiometric ratios. Where ANOVA indicated significant effects were detected, post-hoc comparisons were performed using the Least Significant Difference (LSD) test. Before conducting ANOVA, normality and homogeneity of variance tests were performed on the data. Mantel tests quantified the multivariate correlations between soil properties and soil stoichiometric ratios (C:N, C:P, and N:P). Principal component analysis (PCA) was then conducted to derive composite soil quality scores, enabling comparisons among vegetation types within the Oujiang Estuary and across different *K*. *obovata* forest sites.
